# miR-3678-3p Promotes Esophageal Squamous Cell Carcinoma Progression by Regulating Phosphatase and Tensin Homolog

**DOI:** 10.5152/tjg.2026.25836

**Published:** 2026-04-27

**Authors:** Jinjun Zhang, Ci Cheng, Taiyu Li

**Affiliations:** 1 State Key Laboratory of Chinese Medicine Modernization, Tianjin University of Traditional Chinese Medicine, Tianjin, China; 2 Department of Medical Technology, Nanjing Vocational Health College, Nanjing, China; 3 Department of Oncology, Jiyang People's Hospital of Jinan, Jinan, China

**Keywords:** Biomarker, Esophageal squamous cell carcinoma, miR-3678-3p, PTEN

## Abstract

**Background/Aims:**

Esophageal squamous cell carcinoma (ESCC) is a gastrointestinal malignancy characterized by high invasiveness. The current study aimed to evaluate the role of *miR-3678-3p* in ESCC and its potential mechanisms.

**Materials and Methods:**

The study included 109 ESCC patients. The *miR-3678-3p* expression in ESCC tissues and cells was quantified. The association between *miR-3678-3p* and ESCC severity and adverse prognosis was evaluated. The target relationship between *miR-3678-3p*, phosphatase and tensin homolog (PTEN) was confirmed. The effects of *miR-3678-3p* and *PTEN* on ESCC cell function were analyzed.

**Results:**

ESCC tissues and cells exhibited elevated expression of *miR-3678-3p*. Elevated *miR-3678-3p *expression was significantly associated with malignant clinical features in ESCC, including larger tumor size, advanced tumor–node–metastasis staging, higher lymph node metastasis, and poor tumor differentiation. High *miR-3678-3p* expression served as a predictor of shorter overall survival. *miR-3678-3p* targeted and regulated* PTEN*. *miR-3678-3p* knockdown significantly suppressed the malignant progression of ESCC cells, whereas silencing of *PTEN* reversed these effects. *miR-3678-3p* and *PTEN* jointly participated in the development of ESCC.

**Conclusion:**

*miR-3678-3p* serves as a promising prognostic marker for predicting the severity and adverse outcomes of ESCC. *miR-3678-3p *promotes ESCC progression by targeting *PTEN*.

**Main Points**

- *miR-3678-3p* was closely related to the progression and severity of esophageal squamous cell carcinoma (ESCC).
- *miR-3678-3p* is a promising prognostic biomarker in ESCC.
- *miR-3678-3p* promoted the malignant progression of ESCC cells.
- *miR-3678-3p* and *phosphatase and tensin homolog* jointly regulate the development of ESCC.

## Introduction

Esophageal cancer (EC) is the ninth most common cancer worldwide and is categorized into 2 principal histological subtypes: esophageal squamous cell carcinoma (ESCC) and esophageal adenocarcinoma (EAC).^1^ Over half of all ESCC cases occur in China, where the disease is associated with a high mortality rate.^2^ Currently, radiotherapy remains the primary treatment modality for ESCC.^3^ However, the prognosis of ESCC remains poor because of factors such as radiation resistance, with a 5-year survival rate below 20%.^4^ Therefore, novel biomarkers and reliable therapeutic targets for ESCC warrant further investigation.

MicroRNAs (miRNAs) play a pivotal role in the prognosis and diagnosis of ESCC and drug delivery for ESCC treatment.^5^ Dysregulated miRNA expression is associated with differentiation, invasion, and metastasis in patients with ESCC.^6^ Studies have shown that *miR-3678-3p* is associated with malignant progression of multiple cancers. For instance, Huang et al^7^ demonstrated that *miR-3678-3p* promotes malignant behavior in hepatocellular carcinoma cells. According to Zhang et al,^8^ upregulation of *miR-3678-3p* in colorectal cancer correlates with reduced overall survival. Although upregulation of *miR-3678-3p* in ESCC has been reported,^9^ its role and specific mechanisms in ESCC warrant further investigation.

miRNA influences tumorigenesis, immune evasion, and metastasis of ESCC by targeting and regulating mRNA.^10^ Numerous studies have disclosed that miRNA–mRNA interactions play important roles in ESCC. For example, Pengjie et al^11^ reported that *miR-378a-5p* inhibits ESCC progression through the downregulation of *APOC1/CEP55*. Yang et al^12^ showed that *miR-106b-5p *promotes the viability of ESCC cells while inhibiting apoptosis by regulating *15-hydroxyprostaglandin dehydrogenase* (*HPGD*). Xue et al^13^ demonstrated that *miR-495-3p *is negatively regulated by *fetal-lethal noncoding developmental regulatory RNA *(*FENDRR*) and can predict malignant progression and poor prognosis in ESCC patients. *Phosphatase and tensin homolog* (*PTEN*) is a target gene of *miR-3678-3p* based on database analysis. The role of *PTEN* in ESCC development is well established. For example, Qiu et al^14^ indicated that certain *PTEN* gene mutations are associated with ESCC susceptibility and poor patient prognosis. Yang et al^15^ reported that *PTEN* mediates macrophage polarization via the *PI3K/AKT *pathway and enhances the malignant behavior of esophageal tumor-associated endothelial cells, thereby promoting angiogenesis in ESCC. Therefore the association and role of miR-3678-3p and PTEN in ESCC progression warrant further investigation.

In the current study, the expression of *miR-3678-3p* in ESCC tissues and cells was investigated. To assess the clinical relevance of *miR-3678-3p*, its expression was correlated with clinical parameters in ESCC. The prognostic value of *miR-3678-3p* in ESCC was evaluated. Furthermore, the regulatory role of *miR-3678-3p* on ESCC cell function and the potential mechanisms were investigated.

## Materials and Methods

### Clinical Samples

A total of 109 paired ESCC and adjacent normal tissue samples were collected from Jiyang People’ Hospital of Jinan. All tissue samples were immediately frozen in liquid nitrogen after excision and stored at −80°C. All samples were thawed only once. RNA quality was evaluated by the Agilent 2100 Bioanalyzer with the RNA Nano 6000 Chip, confirming normal profiles. This research adhered to the ethical guidelines of the Helsinki Declaration and was approved by the Research Ethics Review Committee of Jiyang People’ Hospital of Jinan (Approval no: 2019-1025, Date: July 18, 2019). All participants provided informed consent before enrollment. All patients were followed up for 5 years or until their death, with clinic visits scheduled every 3 months in the first 2 years and every 6 months thereafter. All patients who met the diagnostic criteria for ESCC^16^ and newly diagnosed patients were included in the study. Patients with hepatic or renal dysfunction, other gastrointestinal diseases or tumors, severe cardiovascular or cerebrovascular disease, and other severe infectious diseases or immune system disorders were excluded from the study.

### Cell Culture and Transfection

Human normal esophageal epithelial cells (SHEEs) and 4 ESCC cell lines (TE-1, TE-13, KYSE150, and Eca-109) were obtained from the Cell Bank of the Chinese Academy of Sciences (Shanghai, China). These cells were cultured in Dulbecco’s Modified Eagle Medium (DMEM, Gibco, Gibco-BRL; Grand Island, NY, USA) containing 10% fetal bovine serum (FBS, Gibco-BRL, NY, USA) at 37℃ with 5% CO_2_. The *miR-3678-3p* inhibitors, si-*PTEN*, and their negative controls (NCs) were purchased from GenePharma (Shanghai, China) and transfected into ESCC cells via Lipofectamine 2000 (Thermo Fisher Scientific; Cleveland, OH, USA). The *miR-3678-3p* inhibitor sequence was 5′-CCGGUCCGUACAAACUCUGCAG-3′ and the miR-NC sequence was 5′-UCACAACCUCCUAGAAAGAGUAGA-3′.

### Extraction of Total RNA and Real-time reverse transcription PCR

Total RNA was isolated with TRIzol reagent (Solarbio, Beijing, China). cDNA was synthesized via the TaqMan reverse transcription kit (Thermo Fisher Scientific). The expression level of *miR-3678-3p* was detected by a TaqMan microRNA assay (Applied Biosystems, Foster City, CA, USA). The mRNA level of PTEN was quantified using SYBR Green real-time PCR master mix (Applied Biosystems, Foster City, CA, USA). The primer sequences are as follows: *miR-3678-3p* forward primer: 5′-CTGCAGAGTTTGTACGGACCGG-3′ and the miScript universal primer were used for reverse transcription. PCR conditions were as follows: 95°C for 15 minutes and then 40 cycles of 94°C for 15 seconds, 55°C for 30 seconds, and 70°C for 30 seconds. Next, U6 (forward primer: 5′-GCTTCGGCAGCACATATACTAAAAT-3′; reverse primer: 5′-CGCTTCACGAATTTGCGTGTCAT-3′) and GAPDH (forward primer: CTATAAATTGAGCCCGCAGCCTCC; reverse primer: CCCATGGTGTCTGAGCGATGTG) were used as controls for *miR-3678-3p* and *PTEN*. Their relative expression was quantified through the 2^−∆∆ct^ method.

### Cell Counting Kit-8 Assay

The transfected ESCC cells (1 × 10^4^ cells) were plated in 96-well plates for 24, 48, or 72 hours. Subsequently, 10 µL of CCK-8 was added per well. After a 2-hour incubation at 37°C, cell viability was determined by the CCK-8 kit (Beyotime, Shanghai, China), and optical density at 450 nm(OD450) values were measured using a microplate reader (Thermo Fisher Scientific).

### Transwell Assay

For the invasion assay, the upper chamber was precoated with the diluted Matrigel matrix glue. The transfected ESCC cells of each group were maintained in DMEM and seeded in the upper chambers of Transwell plates. DMEM containing 10% FBS was added to the lower chamber and cultured at 37℃ for 24 hours. The cells that remained in the upper chambers were removed. Then, the cells that adhered to the lower membrane surface were fixed with 4% methanol and stained with 1% crystal violet for 20 minutes. Five random fields per chamber were counted under an inverted microscope (Olympus, Tokyo, Japan). The migration assay followed the same protocol as the invasion assay but without Matrigel coating in the upper chamber.

### Dual-Luciferase Reporter Assay

The wildtype or mutated segments of *PTEN* containing the target sequence of *miR-3678-3p* were designed by GenePharma (China) and inserted into the pGL3 luciferase vector (Invitrogen, Carlsbad, CA, USA). ESCC cells were co-transfected with *PTEN*-WT or *PTEN*-MUT and *miR-3678-3p* inhibitor/inhibitor-NC by Lipofectamine 2000 (Invitrogen, Carlsbad, CA, USA). After 48 hours, cell lysates were harvested, and luciferase activity was measured by the dual-luciferase reporter assay kit (Promega, Madison, WI, USA). Renilla luciferase activity was used for normalization of the relative luciferase readings.

### Statistical Analysis

Data were analyzed using GraphPad Prism 10 (GRAPH PAD Software Inc.; California, USA) and SPSS 23 (IBM SPSS Corp.; Armonk, NY, USA). The association between *miR-3678-3p* expression levels and clinical parameters in patients with ESCC was evaluated using the chi-square test. The prognostic performance of *miR-3678-3p* was examined through Kaplan–Meier and Cox regression analyses. The data met the assumptions of normality and homogeneity of variance. Statistical analysis of intergroup differences used the *t*-test (for 2 groups) or 1-way analysis of variance (ANOVA) test (for 3 or more groups), and cell proliferation data were subjected to 2-way ANOVA test. Continuous variables are described in terms of mean ± SD. The experiments were conducted in at least 3 replicates to confirm reproducibility. Bonferroni’s correction was applied for multiple comparisons.

## Results

### *miR-3678-3p* was Upregulated in Esophageal Squamous Cell Carcinoma

**Figure 1:**
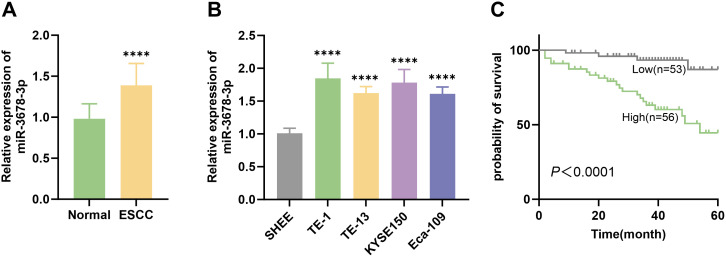
miR-3678-3p expression in ESCC tissues and survival curves. (A) Differential expression of miR-3678-3p in 109 pairs of ESCC and normal tissues. (B) miR-3678-3p is upregulated in 4 ESCC cell lines (TE-1, TE-13, KYSE150, and Eca-109) and SHEEs. (C) High miR-3678-3p expression was associated with shorter survival time in patients with ESCC. ESCC, esophageal squamous cell carcinoma; SHEE, normal esophageal epithelial cells.

The *miR-3678-3p *expression revealed a notable upregulation in ESCC tissues (Figure 1A). In addition, *miR-3678-3p* exhibited increased expression in 4 ESCC cell lines compared to SHEEs (Figure1B).

### Prognostic Significance of *miR-3678-3p* in Esophageal Squamous Cell Carcinoma

Patients with ESCC were divided into low (n = 53) and high *miR-3678-3p* (n = 56) expression groups based on the median tissue expression value. Survival curves showed that high *miR-3678-3p* expression was associated with shorter survival time in patients with ESCC (Figure 1C). Moreover, *miR-3678-3p* expression was significantly associated with tumor size (*P *= .010), tumor–node–metastasis stage (TNM, *P *= .022), lymph node metastasis (LNM, *P *= .031), and tumor differentiation (*P *= .043). However, no significant associations were discovered with age, gender, smoking status, and tumor location (*P *> .05, Table 1). After adjusting for confounding factors, *miR-3678-3p* expression (*P *= .023, Hazard Ratio (HR) = 3.742, 95% CI: 1.203-11.638), tumor size (*P *= .035, HR = 2.476, 95% CI: 1.064-5.763), TNM staging (*P *= .034, HR = 2.475, 95% CI: 1.072-5.713), LNM (*P *= .044, HR = 2.392, 95% CI: 1.022-5.599), and tumor differentiation (*P *= .042, HR = 2.397, 95% CI: 1.032-5.568) remained independent prognostic risk factors in ESCC (Table 2).

### Interaction of *miR-3678-3p* with Phosphatase and Tensin Homolog

**Figure 2:**
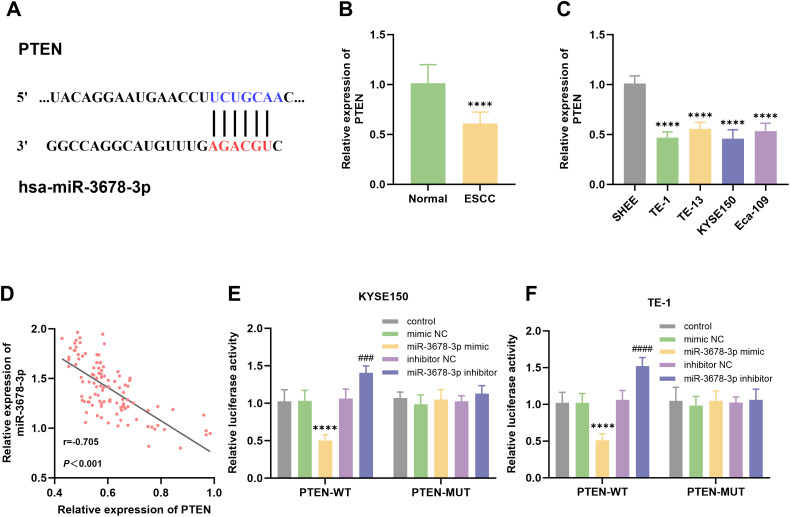
Interaction of miR-3678-3p with PTEN. (A) The binding site between PTEN and miR-3678-3p was predicted using the TargetScan database. (B) PTEN expression is downregulated in ESCC tissues. (C) Decreased level of miR-3678-3p in 4 ESCC cell lines compared with SHEE. (D) The miR-3678-3p expression is negatively correlated with PTEN expression (*r* = −0.705, *P *< .001). (E) and (F) The dual-luciferase assay validated the interaction of PTEN with miR-3678-3p (****P < .0001 vs. normal, SHEE or mimic-NC, ###*P *< .001, ####*P *< .0001 vs. inhibitor-NC). ESCC, esophageal squamous cell carcinoma; NC, negative control; PTEN, phosphatase and tensin homolog; SHEE, normal esophageal epithelial cells.

The predicted binding sequences between *miR-3678-3p* and *PTEN* were identified using the TargetScan database (https://www.targetscan.org/vert_72/). *miR-3678-3p* could directly bind to the 3′ UTR of *PTEN* (Figure 2A). *PTEN* was potently downregulated in ESCC tissues and cells (Figure 2B, C). The *miR-3678-3p* expression (*r* = −0.705, *P *< .001) was negatively correlated with the *PTEN* expression (Figure 2D). The overexpression of *miR-3678-3p* markedly suppressed *PTEN* luciferase activity, whereas its knockdown increased it. However, following a mutation in *PTEN*, the luciferase activity was not significantly affected (Figure 2E, F).

### Effects of *miR-3678-3p* and *Phosphatase and Tensin Homolog* on Esophageal Squamous Cell Carcinoma Cell Function

**Figure 3:**
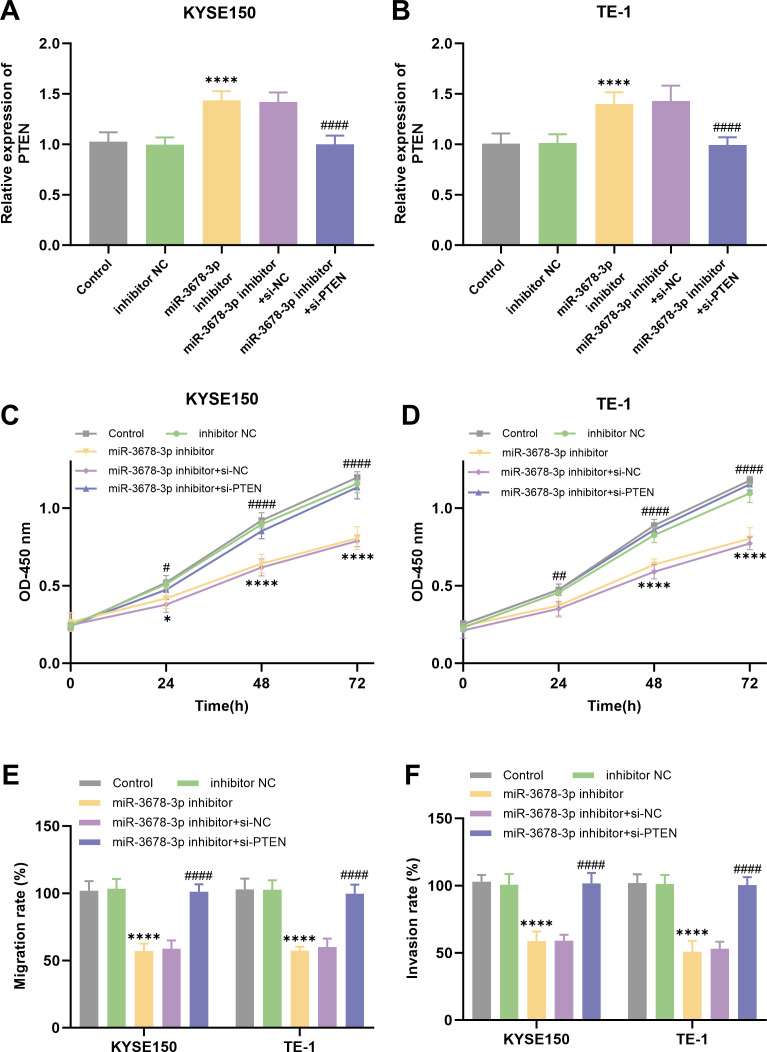
Effects of miR-3678-3p and PTEN on ESCC cell function. (A) and (B) miR-3678-3p knockdown significantly reduced PTEN expression, whereas the effect was reversed by co-transfection of the miR-3678-3p inhibitor and si-PTEN. (C) and (D) The knockdown of miR-3678-3p markedly inhibited the proliferation of ESCC cells, whereas the effect was rescued by knockdown of PTEN. (E)and (F) The knockdown of miR-3678-3p markedly impaired the migration and invasion capacities of ESCC cells, whereas these impacts were effectively counteracted by silencing PTEN (**P < .01, ****P < .0001 vs. inhibitor NC; ^##^*P *< .01, ^####^*P *< .0001 vs. miR-3678-3p inhibitor + si-NC). ESCC, esophageal squamous cell carcinoma; NC, negative control; PTEN, phosphatase and tensin homolog.

*miR-3678-3p* knockdown significantly reduced *PTEN* expression in KYSE150 and TE-1 cells, whereas the effect was reversed by silencing *PTEN *(Figure 3A, B). According to the CCK-8 assay, *miR-3678-3p* knockdown markedly inhibited the proliferation of 2 ESCC cells, whereas the inhibitory effect was effectively counteracted by silencing *PTEN* (Figure 3C, D). Moreover, *miR-3678-3p* downregulation impaired the migration and invasion of ESCC cells via the Transwell assay. However, the silencing of *PTEN* was able to attenuate the impacts (Figure 3E, F).

## Discussion

ESCC is often diagnosed at an advanced stage. Surgical treatment rarely results in complete resection, and recurrence and metastasis are common.^17^ These factors are the primary reasons for the poor prognosis of ESCC. Thus, identifying reliable biomarkers that predict poor outcomes can help forecast treatment outcomes, assist physicians in selecting appropriate treatment plans, and ultimately improve survival rates for patients with ESCC. In the current study, the role and potential mechanisms of *miR-3678-3p* in the progression of ESCC were investigated. The *miR-3678-3p *expression was found to be notably upregulated in both ESCC tissues and cells. Clinically, high *miR-3678-3p* expression was closely associated with malignant clinical features of ESCC, including larger tumor size, advanced TNM staging, elevated LNM rates, and poor tumor differentiation. *miR-3678-3p* was an independent prognostic predictor for ESCC patients, and high *miR-3678-3p* expression was closely associated with reduced overall survival. Functionally, *miR-3678-3p* directly targeted and regulated *PTEN*. Silencing *miR-3678-3p* significantly suppressed the malignant progression of ESCC cells, whereas *PTEN* silencing reversed these effects. This indicates that *miR-3678-3p* and *PTEN* jointly influence ESCC progression.

Numerous studies have demonstrated that miRNA dysregulation, such as *miR-103*, *miR-191-5p*, and *miR-148a-3p*, influences the malignant progression of ESCC.^18^^- 20^ Elevated *miR-3678-3p* expression was observed in ESCC tissues and cells, suggesting a potential oncogenic role. High *miR-3678-3p* expression was significantly linked to larger tumor size, advanced TNM staging, higher LNM rates, and poor tumor differentiation. As traditional clinical prognostic indicators, these factors are closely associated with malignant clinical characteristics in patients with ESCC. Developing personalized treatment plans based on staging facilitates the achievement of superior clinical outcomes. The TNM staging system serves as the primary basis for predicting prognosis and selecting treatment strategies in patients with ESCC.^21^ LNM occurs relatively early in ESCC and is characterized by features such as skip metastasis. Both the metastatic pathways and the number of metastatic sites significantly influence the patients’ overall survival.^22^ Tumor size can improve the prognostic precision of the existing TNM staging system, particularly in node-negative patients.^23^ Poorly differentiated tumors are typically characterized by high malignancy, strong invasiveness, and early metastasis.^24^ Thus, elevated *miR-3678-3p* expression is strongly associated with these indicators, highlighting its potential as a predictor of ESCC severity. Furthermore, *miR-3678-3p* remained an independent prognostic factor for ESCC after adjusting for confounding variables in Cox multivariate analysis. Survival curves revealed that elevated *miR-3678-3p* levels were strongly associated with reduced overall survival in patients with ESCC. Therefore, *miR-3678-3p* represents a promising candidate prognostic biomarker for ESCC. Prognosis assessment is crucial for guiding clinical management and treatment decisions in patients with ESCC. Patients with ESCC with elevated *miR-3678-3p* expression necessitate intensified monitoring and aggressive treatment to improve survival outcomes.

High morbidity and mortality in patients with ESCC stem from its ability to infiltrate and metastasize.^25^ Cell proliferation, migration, and invasion are key cellular processes driving tumor progression and metastasis. Tumor expansion is primarily fueled by the proliferation of cancer cells, whereas migration and invasion mediate the dissemination and metastasis of cancer.^26^ Therefore, understanding the molecular mechanisms underlying ESCC proliferation, invasion, and metastasis is crucial for developing effective therapeutic approaches and improving clinical outcomes. This study demonstrated that *miR-3678-3p* enhances the proliferation, invasion, and migration of ESCC cells, highlighting its crucial role in ESCC progression and metastasis. Furthermore, the possible mechanism by which *miR-3678-3p* promotes ESCC progression was investigated. miRNAs regulate target protein expression through miR–mRNA interactions, thereby exerting pro- or anti-cancer effects.^27^ The database predictions and luciferase assays confirmed that *miR-3678-3p* directly binds to and regulates *PTEN*. Numerous studies have demonstrated that the *PTEN *and miRNA interaction influences the progression of ESCC. For example, Pang et al^28^ showed that plasma exosomal *miR-193a-3p* enhances cellular invasion, migration, epithelial–mesenchymal transition, and metastatic capacity by suppressing *PTEN*. Jia et al^29^ identified *miR-624-3p* as a promoter of ESCC progression by regulating *PTEN*. In the study, co-transfection of si-*PTEN* reversed the functional promotion effect of *miR-3678-3p* on ESCC cells, suggesting that both factors are jointly involved in the progression of ESCC, which might provide a novel therapeutic target in ESCC.

This study elucidates the clinical significance, functional roles, and the molecular mechanisms of *miR-3678-3p* in ESCC. However, this study utilized a relatively small clinical cohort, and the functional investigation of *miR-3678-3p* was conducted solely in vitro. Additional large-scale clinical samples from different pathological grades must be collected, and further animal studies are needed to elucidate its functional role. Research showed that synthetically produced anti-miRNAs have demonstrated encouraging outcomes across multiple cancer types. Anti-miRNAs tightly bind to pathogenic miRNAs overexpressed within cells through base pairing, preventing them from binding to their original target mRNAs. This restores normal cellular function, thereby achieving therapeutic effects.^30^ Consequently, *miR-3678-3p* inhibitors offer a novel therapeutic strategy for ESCC, but their targeted delivery and clinical translation require further investigation. Furthermore, this study preliminarily confirmed the effect of *miR-3678-3p *and *PTEN* on the progression of ESCC; however, the analysis was limited to a single miRNA–mRNA axis. Given the complexity of miRNA regulation, the specific molecular interactions and downstream pathways remain to be fully elucidated.

In conclusion, *miR-3678-3p* is a promising prognostic biomarker that can predict the severity and adverse outcomes of ESCC. Furthermore, *miR-3678-3p* exerts its oncogenic effects by targeting *PTEN*, thereby promoting the progression of ESCC.

## Figures and Tables

**Table 1.** Relationship Between miR-3678-3p Expression and Clinical Characteristics in 109 Patients with ESCC

**Characteristics**	**miR-3678-3p**		**Total** **N = 109**	***P***
	**Low (n = 53)**	**High (n = 56)**		
Age, years				.799
<55	24	24	48	
≥55	29	32	61	
Gender				.676
Male	37	37	74	
Female	16	19	35	
Smoking				.218
Negative	19	14	33	
Positive	34	42	76	
Tumor size (cm)				.010*
<4	40	29	69	
≥4	13	27	40	
TNM stage				.022*
I-II	42	33	75	
III	11	23	34	
LNM				.031*
Negative	43	35	78	
Positive	10	21	31	
Differentiation				.043*
Well + moderate	44	37	81	
Poor	9	19	28	
Tumor location				.637
Middle third of esophagus	27	26	53	
Upper/lower third of esophagus	26	30	56	

ESCC, esophageal squamous cell carcinoma; LNM, lymph node metastasis; TNM, tumor–node–metastasis.

*P < .05.

**Table 2.** Multifactorial Analysis of Cox Regression Affecting the Prognosis of ESCC Patients

**Factors**	***P***	**HR**	**95% CI**
miR-3678-3p expression	.023*	3.742	1.203-11.638
Age	.848	0.920	0.391-2.162
Gender	.152	0.418	0.127-1.379
Smoking	.418	0.689	0.280-1.696
Tumor size	.035*	2.476	1.064-5.763
TNM stage	.034*	2.475	1.072-5.713
LNM	.044*	2.392	1.022-5.599
Differentiation	.042*	2.397	1.032-5.568
Tumor location	.541	1.291	0.570-2.921

ESCC, esophageal squamous cell carcinoma; LNM, lymph node metastasis; TNM, tumor–node–metastasis.

*P < .05.

## Data Availability

The data that support the findings of this study are available on request from the corresponding author.
